# Population Pharmacokinetic Analysis of Cefaclor in Healthy Korean Subjects

**DOI:** 10.3390/pharmaceutics13050754

**Published:** 2021-05-19

**Authors:** Seung-Hyun Jeong, Ji-Hun Jang, Hea-Young Cho, Yong-Bok Lee

**Affiliations:** 1 College of Pharmacy, Chonnam National University, 77 Yongbong-ro, Buk-gu, Gwangju 61186, Korea; rhdqn95@naver.com (S.-H.J.); jangji0121@naver.com (J.-H.J.); 2 College of Pharmacy, CHA University, 335 Pangyo-ro, Bundang-gu, Seongnam-si 13488, Gyeonggi-do, Korea

**Keywords:** cefaclor, population pharmacokinetic, modeling, healthy Korean subjects

## Abstract

The aims of this study were: (1) to perform population pharmacokinetic analysis of cefaclor in healthy Korean subjects, and (2) to investigate possible effects of various covariates on pharmacokinetic parameters of cefaclor. Although cefaclor belongs to the cephalosporin family antibiotic that has been used in various indications, there have been very few population studies on factors affecting its pharmacokinetics. Therefore, this study is very important in that effective therapy could be possible through a population pharmacokinetic study that explores effective covariates related to cefaclor pharmacokinetic diversity between individuals. Pharmacokinetic results of 48 subjects with physical and biochemical parameters were used for the population pharmacokinetic analysis of cefaclor. A one-compartment with lag-time and first-order absorption/elimination was constructed as a base model and extended to include covariates that could influence between-subject variability. Creatinine clearance and body weight significantly influenced systemic clearance and distribution volume of cefaclor. Cefaclor’s final population pharmacokinetic model was validated and some of the population’s pharmacokinetic diversity could be explained. Herein, we first describe the establishment of a population pharmacokinetic model of cefaclor for healthy Koreans that might be useful for customizing cefaclor or exploring additional covariates in patients.

## 1. Introduction

Cefaclor is a β-lactam antibiotic with a mechanism of action similar to penicillin [1]. Cefaclor binds to a specific penicillin binding protein on the cell wall of bacteria [1]. As a result, synthesis of the peptidoglycan layer on the bacterial cell wall is inhibited, leading to cell wall lysis [1]. Peptidoglycans are important components of bacterial cell walls. Human cells do not contain peptidoglycans [2]. Therefore, antibiotics such as cefaclor with mechanisms that can inhibit the synthesis of peptidoglycans have a great advantage by targeting only bacteria without harming human cells. Cefaclor is an antibiotic of the second-generation cephalosporin family. It has antibacterial activities against aerobic Gram-positive microorganisms (such as *Staphylococcus aureus*) and Gram-negative microorganisms (such as *Haemophilus influenzae*) [1]. In addition, cefaclor is effective against certain anaerobic microorganisms (such as *Propionibacteria acnes*). It is a drug of choice as an empirical treatment for many indications due to its effectiveness and tolerability [1]. Cefaclor has a greater bactericidal action than other first- and second-generation cephalosporin antibiotics. It is an alternative drug for patients with penicillin allergy (although there are still some cross-reactivity) [3]. Its clinical use continues from the past to the present [3]. Representative clinical indications for cefaclor include upper and lower respiratory tract infections, pneumonia, acute sinusitis, otitis media, and skin infection [1]. Major reported side effects of cefaclor include itching, hives, rash, diarrhea, and indigestion [1,3]. These side effects are not fatal, unlike some antibiotics such as aminoglycoside type antibiotics. Although cefaclor has been recognized as a relatively safe antibiotic, studies on its individual clinical therapy or dose through population pharmacokinetic analysis and model studies are insufficient. According to past reports, cefaclor is partly metabolized in the liver and excreted mainly through urine (renal excretion) [1,4,5]. Therefore, if there is a problem with liver or renal function (especially in the case of dialysis), the prescribed dose of cefaclor needs to be adjusted and caution should be exercised [1]. However, scientific studies on the degree of precise correlation between individual physiological or biochemical factors in the pharmacokinetics of cefaclor and the necessity of dose adjustment through these factors are insufficient.

Although a population pharmacokinetic model for cefaclor in Chinese has been reported [6], the study has only enrolled 20 healthy Chinese men, thus having limitations in presenting major covariates that can influence individual pharmacokinetics of cefaclor. The influence of cranberry juice on the pharmacokinetics of cefaclor has been studies and a population pharmacokinetic model for cefaclor has been introduced [7]. However, it was a population pharmacokinetic model of 18 adult women with only age and weight of subjects as covariates. Above all, the presentation of objective judgment data (including model building steps) to confirm the improvement of the model was insufficient and model validation was not performed. Therefore, these previously reported models could not be directly applied to the setting of individual clinical therapy for patients.

To date, basic data for determining the dosage regimen for cefaclor-administered patients in clinical practice are lacking and most dosages are empirical. That is, there has been little research that precisely finds covariates such as physiological and biochemical parameters that can influence pharmacokinetic parameters of cefaclor in healthy adults through population pharmacokinetics. Therefore, the objectives of this study were: (1) to analyze population pharmacokinetics of cefaclor in healthy Korean adult males, and (2) to obtain basic data applicable to patients receiving cefaclor in the future such as a phase I study.

Cefaclor is one of the most frequently prescribed drugs in Korea. Its usage is about 1.5 times higher than the Organization for Economic Co-operation and Development (OECD) average for antibiotic use [8]. However, dose-setting studies considering the differences between individuals compared to the clinical significance and use of cefaclor were very lacking. A population pharmacokinetic model of cefaclor for Koreans is urgently needed. Considering the necessity and importance of such studies, we analyzed population pharmacokinetics of cefaclor in Koreans. By developing a population pharmacokinetic model that takes into account intra- or inter-individual variability of cefaclor, effects of inter-individual factors on cefaclor pharmacokinetics can be evaluated. As a result, individual pharmacokinetic parameter values of cefaclor can be more accurately estimated, enabling personalized clinical therapy. In other words, by exploring major covariates through analysis of population pharmacokinetics and model study of cefaclor in a clinic setting, it will be possible to set the dose and regimen to maximize the therapeutic effect while reducing side effects of cefaclor for patients.

## 2. Subjects and Methods

### 2.1. Subjects

Samples from cefaclor bioequivalence studies of healthy Korean males for population pharmacokinetic analysis of cefaclor were included in this study (as a retrospective approach). Only data from the reference formulation (Ceclor^®^ capsule, Lilly Korea Co., Ltd., Seoul, Korea) were used for this population pharmacokinetic analysis. The total number of subjects used in the analysis was 48. Their age, weight, and height were 19 to 26 years, 50.0 to 88.7 kg, and 164.0 to 187.3 cm, respectively. Their body surface area (BSA) and body mass index (BMI) were 1.51–2.10 m^2^ and 17.70–28.44 kg/m^2^, respectively. BSA was calculated using the Mosteller equation [9]. BMI was calculated based on the Kaup index [10]. Both BSA and BMI were determined using calculations generally applied to adults. 

No subject had any previous medical history or drug hypersensitivity reaction. All subjects were physically normal (which means the subjects were healthy enough to participate in the bioequivalence study of cefaclor judged by physician). All subjects provided prior written consent to conduct bioequivalence and pharmacokinetic studies. All subjects underwent a physical examination, clinical screening, total blood count, urine test, and blood chemistry analyses before participating in this clinical study to assess their physical health. Subjects were excluded from this study if they had taken other drugs or alcohol for at least one week prior to this clinical study. This study protocol was thoroughly reviewed and approved by the Institutional Review Board of the Institute of Bioequivalence and Bridging Study, Chonnam National University, Gwangju, Republic of Korea (bioequivalence study permit numbers: 112; 9 March 2004, and 121; 9 August 2006). Clinical studies were conducted in accordance with the Rules of Good Clinical Practice and the revised Declaration of Helsinki for biomedical research on human subjects. Bioequivalence studies were performed as randomized, single-dose, open-label, crossover, and two-way studies with a washout period of 7 days.

### 2.2. Sampling

Prior to the clinical trial, each subject had a heparin-locked catheter installed in the vein of the arm or the back of the hand and 5 mL of blank blood was collected. Subsequently, subjects were given a single dose of cefaclor capsules (250 mg) orally with 240 mL of water. Blood sampling time from subjects were as follows: before administration (0 h) and at 0.25, 0.5, 0.75, 1, 1.25, 1.5, 2, 2.5, 3, 4, and 5 h after oral administration. The sampling method was as follows: at the time of blood collection, approximately 2 mL of blood was drained each time to completely remove the heparinized saline solution remaining in the venous catheter. After that, about 5 mL of blood was collected and placed in Vacutainer tubes with the subject management number and blood collection time. After each blood collection, physiological saline solution for injection with heparin was injected to prevent clotting of the blood remaining in the intravenous catheter. The blood in the Vacutainer tube was centrifuged at 3000× *g* for 20 min. The plasma was separated from the supernatant into polyethylene tube and stored at −80 °C until analysis.

### 2.3. Determination of Clinical Biochemistry Parameters

The following clinical biochemical parameters were determined for population pharmacokinetic analysis of cefaclor: albumin, total proteins, blood urea nitrogen (BUN), total bilirubin, cholesterol, alanine transaminase (ALT), aspartate transaminase (AST), albumin, alkaline phosphatase (ALP), creatinine, and creatinine clearance (CrCl). CrCl was calculated using the commonly used Cockcroft–Gault equation [11]. Clinical biochemical parameters were determined by plasma analysis using a dry automatic analyzer of microsides VITROS (Ortho Clinical Diagnostics, Raritan, NJ, USA) operating with a reflectance spectrophotometer (Korea Process Technology Co., Ltd., Seoul, Korea).

### 2.4. Determination of Plasma Cefaclor Concentrations

Plasma concentrations of cefaclor were determined using high performance liquid chromatography–ultraviolet (HPLC–UV) assay established and validated in a previous study [12].

#### 2.4.1. Chromatographic Conditions

The main equipment used in the analysis was as follows: Shimadzu LC 10 ADvp System (Shimadzu Inc., Kyoto, Japan) consisting of an LC-10ADvp pump, a DGU-12A degasser, a CTO-10Avp column oven, an SCL-10Avp system controller, and an SPD-10Avp UV detector. System operation and data processing were performed using Shimadzu Model Class LC-10 system software (Shimadzu Inc.). The stationary phase used for chromatographic separation was a Symmetry C18 column (Waters Corp., Milford, MA, USA) with the following specifications: an inner diameter of 4.6 mm, a length of 150 mm, and a particle size of 5 µm. A mixed solution of 88:12 (*v*/*v*) water and methanol as the mobile phase was used at a flow rate of 0.8 mL/min. The temperature of the column was kept at 30 °C. Quantification of cefaclor was performed at a wavelength of 265 nm.

#### 2.4.2. Calibration Curve

To quantify cefaclor in the sample, a calibration curve was prepared using an internal standard. The preparation of standard samples for the calibration curve was carried out with the following procedures. The cefaclor standard was dissolved in water to a concentration of 1 mg/mL. It was then diluted and added to blank human plasma to prepare a standard plasma solution for calibration curve so that the concentration of cefaclor in the final plasma was in the range of 0.1 to 10 µg/mL. To 500 µL of each standard plasma solution, 25 µL of an internal standard (cephradine of 50 µg/mL) solution and 500 µL of 6% (*v*/*v*) trichloroacetic acid were added. Cephradine as the internal standard was dissolved in methanol. Standard plasma liquids with organic solvents added were vortexed for 30 sec and then centrifuged at 10,000× *g*. The supernatant (50 µL) was then injected into the HPLC system using a Rheodyne injector (Shimadzu Inc.). The calibration curve was prepared with the ratio of the peak area of cefaclor to the peak area of the internal standard as the *y*-axis and the theoretical concentration of cefaclor as the *x*-axis.

#### 2.4.3. Sample Preparation

Plasma samples stored at −80 °C after sampling at each predetermined time from subjects were thawed at room temperature (25 °C) and stirred for 3 s. Then 500 µL of plasma sample was mixed with 25 µL of an internal standard solution (cephradine at 50 µg/mL) and 500 µL of 6% (*v*/*v*) trichloroacetic acid and pretreated in the same manner as mentioned to prepare the calibration curve. The volume of sample injection into the HPLC system was 50 µL, which was the same as mentioned in the calibration curve. The concentration of cefaclor in the sample was calculated by substituting the ratio of the peak area of cefaclor to the peak area of the internal standard to the calibration curve.

#### 2.4.4. Analytical Validation

The HPLC–UV method used to quantify the concentration of cefaclor in each plasma sample has already been well validated in a previous study [12]. Verification items included selectivity, specificity, linearity, precision, accuracy, carryover, recovery, dilution integrity, stability, and incurred sample reanalysis.

### 2.5. Pharmacokinetic Analysis

Base pharmacokinetic parameters of cefaclor were subjected to a non-compartmental analysis using Phoenix WinNonlin software version 8.3 (Pharsight, Certara Inc., Princeton, NJ, USA). The area under the curve from 0 h to infinite (AUC_0–∞_) was calculated as the sum of AUC_0–t_ and C_last_/k, where C_last_ was the last measurable concentration, t was the time in C_last_, and k was the elimination rate constant at terminal phase. AUC_0–t_ was calculated using a linear trapezoidal rule from 0 to t h after oral administration. The half–life (T_1/2_) was calculated as 0.693/k and the volume of the distribution (V/F) was calculated as dose/k × AUC_0–∞_. The clearance (CL/F) was calculated by dividing the dose (as 250 mg) of cefaclor by AUC_0–∞_, where F was the bioavailability of oral administration. Quantification results of cefaclor obtained using HPLC–UV were plotted as graphs of cefaclor plasma concentration (*y*-axis) over time (*x*-axis). The highest drug concentration in the plasma (C_max_) and the time to reach C_max_ (T_max_) were determined from the plasma cefaclor concentration–time curve of each individual. All pharmacokinetic parameter values were estimated as mean ± standard deviation (SD).

### 2.6. Model Development

Construction and analysis of cefaclor’s population pharmacokinetic model was performed using a non-linear mixed effects model with Phoenix nonlinear mixed effects model (NLME) software version 8.3 (Pharsight, Certara Inc.). Population pharmacokinetic analysis of drugs using this program has been actively performed [13,14]. Population pharmacokinetic model development for cefaclor was performed in the first-order conditional estimates method with extended least squares estimation (with ŋ–*ε* interaction). To construct and analyze the population pharmacokinetic model of cefaclor, three steps were used. The first step was to establish a base model that could well explain the pharmacokinetics of cefaclor. The second step was to explore significant covariates that could account for the pharmacokinetic variability of cefaclor between individuals. The third step was to finally establish a population pharmacokinetic model that could well explain overall pharmacokinetic results of cefaclor obtained experimentally by applying explored covariates to the base model of cefaclor established in the previous step.

To establish a suitable compartment model as the base model structure, data of cefaclor concentration in plasma over time of 48 subjects were applied to various compartment models, including one-, two-, and three-compartment disposition models. Regarding the transit rate of cefaclor between compartments, zero-order absorption/elimination, first-order absorption/elimination, and Weibull absorption were attempted. Oral absorption of cefaclor with or without absorption lag-time and multiple transit models were tried. The final selection of a structural base model was performed based on the statistical significance between models using Akaike’s information criterion (AIC), goodness-of-fit plots, and twice the negative log likelihood (−2LL). Changes in statistical significance according to an increase or decrease in the number of parameters used in the model were also considered. 

Additive, log-additive, proportional, mixed, and power error models were tried as models to explain residual variability. The inter-individual variability (IIV) in pharmacokinetic parameters of cefaclor were evaluated using an exponential error model as shown in the following equation: P_i_ = P_tv_ × exp(ŋ_i_), where ŋ_i_ was the random variable for the ith individual, which was normally distributed with mean 0 and variance ω^2^, P_i_ was the parameter value of the ith individual, and P_tv_ was the typical value of the population parameter. It was confirmed that considering IIV in each parameter had a major effect on model improvement. Collected physiological and biochemical parameters were applied to explore potential covariates that could explain the pharmacokinetic variability of cefaclor between individuals. That is, the degree of correlation between each individual’s pharmacokinetic parameters and physiological or biochemical parameters was first screened through regression analysis. Selected candidate covariates were then sequentially applied to the IIV model. The effect of each covariate was checked using exponential or additive or power options. By stepwise backward elimination and forward addition procedure, covariates were included or eliminated. By change in the objective function value (OFV), the inclusion of covariates was determined. Covariates corresponding to a decrease in the OFV value greater than 3.84 (*p* < 0.05) were included in the base model (in the forward addition procedure). Covariates corresponding to the case where the decrease in OFV value was greater than 6.63 (*p* < 0.01) through the backward elimination process were also included in the model.

### 2.7. Model Evaluation

The final established cefaclor population pharmacokinetic model was evaluated and validated visually or numerically. All processes of model evaluation and validation were performed using Phoenix NLME and R software (R Core Team). Evaluation of the model was largely performed through the following four methods: goodness-of-fit (including distribution of residuals), visual predictive check, bootstrapping, and normalized prediction distribution error. The goodness-of-fit was confirmed using diagnostic scatter plots as follows: (A) population-predicted concentrations (PRED) versus observed (DV), (B) individual-predicted concentrations (IPRED) versus DV, (C) PRED versus conditional weighted residuals, (D) time (IVAR) versus conditional weighted residuals, and (E) quantile–quantile plot of components of conditional weighted residuals. By using the visual predictive check option of Phoenix NLME, visual predictive check of the final established model was performed. The number of simulations for the visual predictive check was 1000. IVAR–DV concentration data were graphically superimposed on median values and the 5th and 95th percentiles of IVAR–simulated concentration profiles. If DV concentration data were approximately distributed within the 95th and 5th prediction interval, the model was expected to be precise. Using non-parametric bootstrap analysis, stability of the final model was confirmed and bootstrap option of Phoenix NLME was used. A total of 1000 replicates were generated by repeated random sampling with replacement from the original dataset. Estimated parameter values such as standard errors (SE, including confidence intervals) and medians from the bootstrap procedure were compared with those estimated from the original dataset. Normalized prediction distribution error was used to evaluate the predictive performance of the model based on a Monte Carlo simulation with the R package. Normalized prediction distribution error results were summarized graphically using (A) quantile-quantile plot of the normalized prediction distribution error, (B) a histogram of the normalized prediction distribution error, (C) scatterplot of normalized prediction distribution error versus IVAR, and (D) scatterplot of normalized prediction distribution error versus PRED. If the predictive performance was satisfied, normalized prediction distribution error would follow a normal distribution (Shapiro–Wilk test) with a mean value of zero (*t*-test) and a variance of one (Fisher’s test).

## 3. Results and Discussion

### 3.1. Study Design and Demographic Analysis

Bioequivalence test results (from reference formulation) performed on 48 healthy Korean males were used to analyze cefaclor’s population pharmacokinetic model. As a result, a total of 521 cefaclor plasma concentrations were available for population pharmacokinetic modeling. All physiological information such as age, weight, and height for 48 bioequivalence test participants were obtained. In addition, as mentioned above, albumin, total protein, BUN, total bilirubin, cholesterol, ALT, AST, ALP, and creatinine levels of participants were obtained through biochemical analysis of plasma samples. Related demographic characteristics of participants are shown in Table 1.

### 3.2. Determination of Plasma Cefaclor Concentrations

As mentioned earlier, an analysis method validated in a previous study [12] was applied in this study. The run time per sample of the HPLC–UV method used to determine the concentration of cefaclor from plasma samples was 25 min. The equation of the mean calibration curve for cefaclor obtained from plasma samples was as follows: Peak area ratio of cefaclor and internal standard = 0.450 × concentration of cefaclor + 0.0197, showing an excellent linearity in the range of 0.1 to 10 µg/mL. Correlation coefficient values of the calibration curve were very high (above 0.99). Cephradine was used as an internal standard because it was structurally very similar to the analyte cefaclor. Thus, it was suitable in terms of physicochemical properties related to chromatographic separation. There were no significant interferences derived from system or endogenous substances peaks, and it was confirmed the identical cefaclor peak spectrum with the UV detector. Intra–batch (*n* = 5) accuracies for cefaclor ranged from 96.35% to 104.42%, with precision (coefficient of variation, CV) < 6.34%. Inter–batch (*n* = 5) accuracies for cefaclor ranged from 95.48% to 106.27% with precision (CV) < 6.53%. There was no carryover phenomenon. Analyte remaining during the analysis that might appear in the next analysis was not confirmed. Recoveries for both cefaclor and internal standard were consistently higher than 90%. Stabilities of cefaclor in plasma samples under various conditions (including short-term, long-term, freeze-thaw, and autosampler stabilities) were all within 100 ± 15%. After diluting cefaclor to a concentration of 10 µg/mL or more with blank plasma, analysis results did not deviate more than 20% from the theoretical concentration value. As a result of reanalysis, differences in quantitative values of cefaclor were all within 15%, proving the reproducibility of the method. As a result, the HPLC–UV method used in this study was sufficiently reverified in terms of selectivity, specificity, linearity, precision, accuracy, carryover, recovery, dilution integrity, stability, and incurred sample reanalysis, a major step for accurate pharmacokinetic studies.

### 3.3. Pharmacokinetic Results by Non-Compartmental Analysis

The rationale for setting blood sampling times was as follows. Reported cefaclor blood T_1/2_ ranged from approximately 0.5 to 1.5 h [1,4,5,7,12,15,16,17,18,19,20,21,22,23,24,25,26]. Therefore, blood sampling time after dosing was set to be 5 h, which was at least 3 times T_1/2_. The number of sampling was set to at least 2 points before reaching C_max_. Blood sampling was performed at 0.25 h intervals up to 1 h after dosing, taking into account that the reported T_max_ of cefaclor was within approximately 1 h [1,4,5,7,12,15,16,17,18,19,20,21,22,23,24,25,26]. In addition, taking into account the preliminary information [1,4,5,7,12,15,16,17,18,19,20,21,22,23,24,25,26] that cefaclor’s total loss from blood was large and relatively rapid, blood sampling was continued at 0.25, 0.5, or 1 h intervals until 5 h after administration. Pharmacokinetic results of previous studies (in Koreans) are summarized in Table S1. Pharmacokinetic results of cefaclor in healthy adults other than Koreans presented in past studies are summarized in Table S2. As shown in Tables S1 and S2, a number of pharmacokinetic studies of cefaclor have been performed since the past. Pharmacokinetic results for cefaclor in previous studies [1,4,5,7,12,15,16,17,18,19,20,21,22,23,24,25,26] were not significantly different from those of the present study. That is, the pharmacokinetic parameter values obtained from this study were within the overall range (without much deviating) of the values presented in Tables S1 and S2 at the same dose.

**Figure 1:**
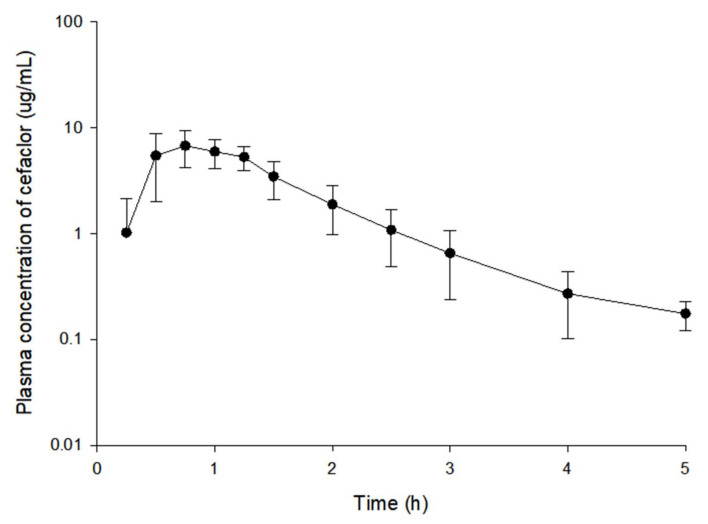
Log-transformed mean plasma concentration–time profiles of cefaclor. Vertical bars represent standard deviation of the mean (*n* = 48).

The observed plasma concentration (log scaled)–time profiles of cefaclor in 48 subjects after oral administration at 250 mg dose are presented in Figure 1. Plasma concentration values in the oral absorption phase of cefaclor varied greatly among individuals. In particular, the diversity of cefaclor plasma concentrations between individuals at 0.25 and 0.5 h after oral administration was significant. In addition, C_max_ of cefaclor was reached within 0.5–1.5 h after oral administration. Cefaclor concentration in the plasma rapidly decreased thereafter (shown in Figure 1). After oral administration of cefaclor, quantification values from several subjects corresponded to below the limit of quantification (BLOQ) at 0.25 h, the first point of sampling and 5 h, the last point of sampling. Considering the relatively high lower limit of quantification (LLOQ) value of 100 ng/mL, the BLOQ samples were treated as missing values.

Pharmacokinetic parameter values calculated by non-compartmental analysis are presented in Table 2. The average T_1/2_ value was very short at 0.68 h and the T_max_ was fast at 0.80 h, consistent with previously reported pharmacokinetic results of cefaclor in healthy adults [1,4,5,7,12,15,16,17,18,19,20,21,22,23,24,25,26]. In addition, a very large CL/F value of 27188.46 mL/h suggested a widespread loss of cefaclor from blood, consistent with previous reports [7,15,22,23]. The AUC_0–t_/AUC_0–∞_ ratio (%) was over 80% and the mean C_max_ value of cefaclor was high at 7.87 µg/mL. Taking these results into account, a LLOQ at 0.1 µg/mL suggested that it was sensitive enough to conduct pharmacokinetic studies with plasma samples obtained after oral administration of cefaclor to humans. It also implied that the setting of sampling points was appropriate. Nevertheless, considering that the plasma drug concentration became BLOQ at 0.25 h or 5 h after oral administration of cefaclor from several individuals, a more sensitive assay (with a lower LLOQ) will need to be applied to the pharmacokinetic study of cefaclor in the future. Pharmacokinetic parameter values obtained from non-compartment analysis were used as initial values in constructing the base model of cefaclor’s population pharmacokinetic.

### 3.4. Population Pharmacokinetic Model Analysis

Plasma concentration data obtained after oral administration of cefaclor 250 mg to humans were well explained as one-compartment among the compartmentalized base models. When applied with mean plasma concentration–time data, the two-compartment model showed a better graphical fit and lower AIC than the one-compartment model. However, when applying the model to each of 48 subjects, the one-compartment model was applicable to all subjects whereas the two-compartment model was only applicable to 35 individuals. In other words, it was not possible to calculate optimal model parameters with the best fit for 13 individuals in the two-compartment model. This implied that plasma concentration–time data of cefaclor obtained in this study were better explained with the one-compartment model than with the two-compartment model. Therefore, the one-compartment model was considered as the base compartment model structure. Trials were also performed with three-compartment models. However, they were unsuitable for explaining plasma concentration–time data of cefaclor. In a similar study reported previously [14], multiple transit compartment models have also been tried. However, models an increasing number of parameters did not show significant improvement. Improved goodness-of-fit was identified with lower AIC and −2LL values given for both one- and two-compartments than when there was no lag-time. This implied that a model with a lag-time in the absorption phase was more suitable as a pharmacokinetic model of cefaclor. Rates of absorption and removal of cefaclor from the compartment were better described with the first-order than with the zero-order. Weibull absorption model applied in a previous study [7] was also tried in this study. However, the model improvement was not significant compared to an increase in the number of parameters. As a result, pharmacokinetic parameters used in the base structure model were as follows: clearance for the central compartment (CL/F), volume of distribution for the central compartment (V/F), first-order oral absorption rate constant (K_a_), and absorption lag-time (T_lag_). The base model of cefaclor established in this study was the same as in the on in a previous study [26]. That is, according to Oguma et al. [26], plasma concentration profiles of cefaclor were well fitted to a conventional one-compartment model with a first-order absorption process. In the pharmacokinetic model of cefaclor reported previously [6], two-compartment (with first-order absorption) was set as the base model. The reason for the difference in the number of compartments compared to the base model chosen in this study was probably due to the analysis of a limited number of people (*n* = 20). In another previous study [7], one-compartment was selected as the base model of cefaclor, the same as in the current study. This implied that the one-compartment as a base model structure of cefaclor established in this study could be properly considered without any major problems.

As a result of searching for a suitable residual error model, it was confirmed that AIC and −2LL values were significantly lowered in the proportional error model. This suggested that the proportional residual error model was a suitable model to account for cefaclor’s intra–individual variability. When the residual error model was used as a log additive, ω shrinkage values of V/F and CL/F were very large (over 0.99). Thus, it was not a suitable residual error model. For CL/F, V/F, K_a_, and T_lag_ parameters used in the base model, IIV was sequentially considered or excluded to determine whether the exponential error IIV model was appropriate. As a result, it was confirmed that the model was significantly improved with a reduction in the total number of estimated parameters in the model considering IIV for parameters of CL/F, V/F, and K_a_. T_lag_ was considered to have a typical value (tv). Table 3 summarizes steps used to develop the base structural model of cefaclor mentioned so far. In addition, cefaclor plasma concentration data were converted to a log scale to construct a new data set. The same model building processes mentioned earlier were applied. However, no significant model improvement was confirmed.

**Figure 2:**
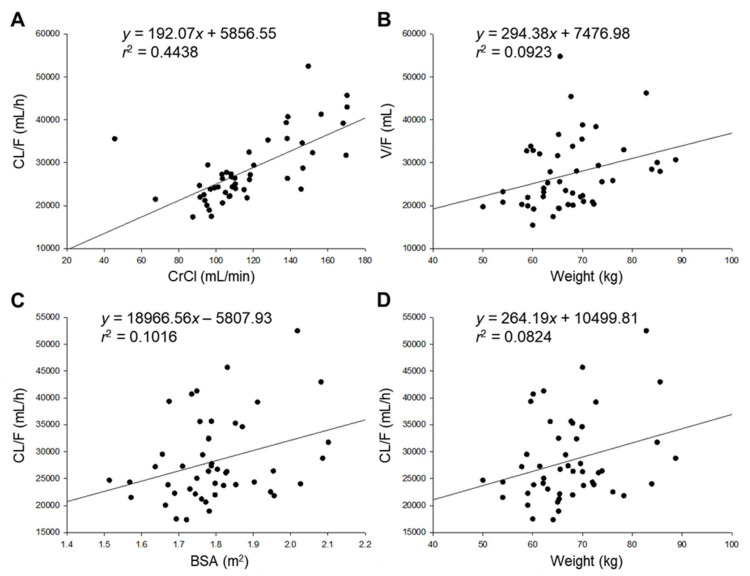
Relationship between subjects’ characteristics and individual predicted pharmacokinetic parameters. CL/F of cefaclor according to CrCl (**A**), V/F of cefaclor according to weight (**B**), CL/F of cefaclor according to BSA (**C**), and CL/F of cefaclor according to weight (**D**).

In order to explore covariates that could explain the variability in pharmacokinetic parameters of cefaclor between individuals, pharmacokinetic parameters obtained by non-compartment analysis were primarily correlated with collected potential covariates. These potential covariates refer to physical and biochemical characteristics of subjects mentioned earlier. Figure 2 shows a graph by plotting potential covariates against individual post–hoc parameters. Covariates showed a relatively high (Figure 2A) or weak (Figure 2B–D) degree of correlation with pharmacokinetic parameters. CrCl is often used as an indicator of renal function. It showed a high positive correlation with CL/F. This is closely related to the information that cefaclor is excreted primarily through kidneys and urine [1]. Another similar study has reported a positive linear correlation between elimination rate constant of cefaclor (from blood) and CrCl [5]. In addition, there were positive correlations between body weight and V/F, between body weight and CL/F, and between BSA and CL/F. This was related to the report that the activity of cytochrome P450 and phase II conjugation related to drug metabolism is increased with weight gain [27]. In addition, correlations between potential covariates and parameters used in the cefaclor population pharmacokinetic model shown in Figure 2 have also been reported in studies on pharmacokinetic analysis and modeling of other drugs [13,14]. Figure S1 shows graph results of those whose degrees of correlation were confirmed after those shown in Figure 2. Although the degree of correlation was not large, a small positive correlation was found between BMI and V/F. A small positive correlation was also found between BMI and CL/F. This implies that when BMI as an indicator of obesity increases, the volume of distribution and removal rate of cefaclor will also increase. These correlations were similar for other drugs previously reported [27]. Significant correlations between other demographic characteristics and pharmacokinetic parameters have not been identified, and these have been difficult to explain. The influence of each selected candidate covariate on pharmacokinetic parameters of cefaclor was evaluated by sequentially applying covariates to the base structural model established in the previous step. Physiological and biochemical parameters applied in this study were normalized to the median (of the observed values) as a continuous covariate. These medians were reflected in the model. The fit of the model was judged by a change in the OFV value compared to cefaclor’s base model (without covariates). When weight, BSA, and BMI were included in the base model of cefaclor as covariates of CL/F and V/F, OFV was slightly reduced, but the decrease in the OFV was lower than the significance criterion of 3.84 (*p* < 0.05). Although body weight was considered an effective covariate of CL/F in a previously reported Chinese cefaclor population pharmacokinetic model [6], it was not included as an important covariate in the current cefaclor population pharmacokinetic model for Koreans. However, when CrCl was included in the base model of cefaclor as a covariate of CL/F, an OFV reduction of 4.761 was found. Therefore, CrCl was considered an effective covariate of CL/F instead of body weight. This implied that the dose and administration of cefaclor could be adjusted according to the degree of renal function, making it possible to have individual customized medications in the future. Previous studies have shown that cefaclor loss from blood in patients with impaired renal function is 5–10 times slower than that in normal subjects [4,5]. It was very interesting to see a significant correlation in CL/F of cefaclor according to renal function, although our study was on healthy adults. According to a previous report, cefaclor and cefprozil were mostly excreted in the urine through the kidney [20]. A correlation between CrCl and CL/F was confirmed in a population pharmacokinetic model of cefprozil for healthy adults, and that correlation was considered the main covariate [13]. For cefaclor, the correlation between CrCl and CL/F was reflected as the main covariate, similar to that found for cefprozil. This result did not deviate significantly from the existing fact. Although clinical side effects of cefaclor are less severe than those of other high–risk antibiotics, skin and digestive side effects are frequently reported [1]. Considering both side effects and sufficient drug concentration to obtain effective therapeutic effects of cefaclor, it is important to maintain the therapeutic effect while reducing side effects of cefaclor by lowering the dose for those with low renal function. In addition, when body weight was included in the base model of cefaclor as a covariate of V/F, an OFV reduction of more than 3.84 (*p* < 0.05) was confirmed. Thus, weight was considered as an effective covariate of V/F, consistent with a previous report for a population pharmacokinetic model of cefaclor in 18 healthy women [7] that confirmed the correlation between body weight and V/F as a covariate. The present study also found a correlation between body weight and V/F in the population pharmacokinetic model for cefaclor. However, specific values for the degree of correlation between weight and V/F were not presented and validations of the model were not performed in the previous study [7]. Thus, efforts were made to compensate for these limitations in the present study.

As a result, when CrCl was considered as a covariate of CL/F and weight as a covariate of V/F, OFV, an index of model fit, was reduced more than 6.63 (*p* < 0.01), which was the backward elimination criterion. Therefore, CrCl and body weight were selected as effective covariates for the final cefaclor population pharmacokinetic model. Table 4 summarizes the covariate selection process for the final population pharmacokinetic model of cefaclor according to OFV. Other candidate covariates such as BSA, BMI, AST, ALT, ALP, BUN, and total protein were not valid for model improvement due to inter-individual pharmacokinetic diversity of cefaclor. Although all candidate covariates were attempted to search for associations with CL/F and V/F as well as K_a_ and reflect the model, no additional significant associations were identified. In this regard, more covariates need to be identified in the future through genotyping of transporters (such as *PEPT1*) related to oral uptake of cefaclor. A previous study has confirmed that cefaclor is a major substrate for human peptide transporter–1 (hPepT1) [28]. Therefore, it is necessary to explore potential covariates that can further explain the pharmacokinetic diversity of cefaclor by applying information on the *PEPT1* gene related to the activity and expression of hPepT1.

**Figure 3:**
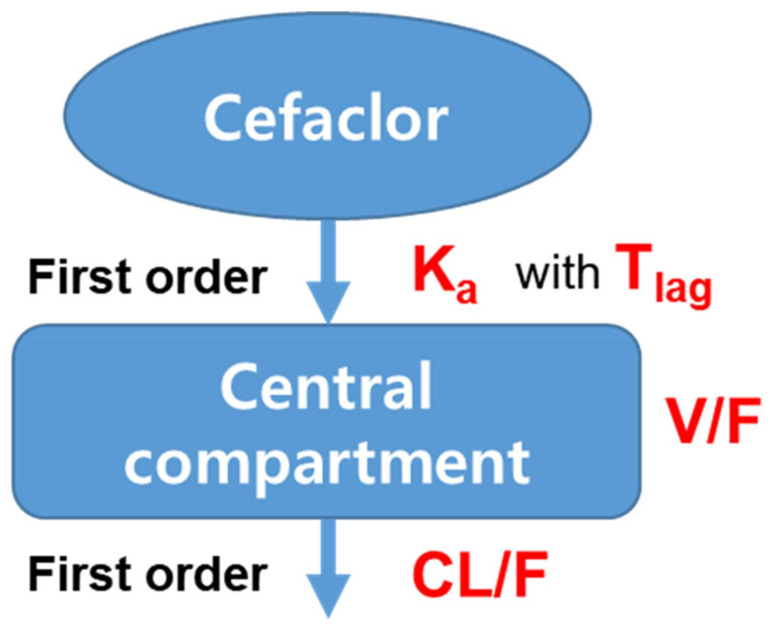
Population pharmacokinetic model structure of cefaclor.

The structure (shown in Figure 3) and equation of the final established cefaclor population pharmacokinetic model reflecting effects of covariates are as follows:

V/F = tvV/F · ((Weight/66.05) ^ dV/FdWeight) · exp(*ŋ*_V/F_) CL/F = tvCL/F · ((CrCl/110.92) ^ dCL/FdCrCl) · exp(*ŋ*_CL/F_) T_lag_ = tvT_lag_ K_a_ = tvK_a_ · exp(*ŋ*_Ka_)

In this formula, dV/FdWeight and dCL/FdCrCl represent the degree of correlation between body weight and V/F, and CrCl and CL/F, respectively.

Parameter values estimated by cefaclor’s population pharmacokinetic model are presented in Table 5. These pharmacokinetic parameter values estimated from the finally established population pharmacokinetic model of cefaclor were not significantly different from those estimated by non-compartmental analysis. For example, tvV/F and tvCL/F values calculated with the population pharmacokinetic model were 22,593.26 and 27,166.88 mL/h, respectively, and V/F and CL/F values calculated by non-compartmental analysis were 26,116.39 ± 7820.88 mL (mean ± SD) and 27188.46 ± 7771.88 mL/h (mean ± SD), respectively. These values did not differ by more than 20% from each other, indirectly implying that the final established population pharmacokinetic model of cefaclor described experimentally obtained pharmacokinetic results relatively well. In the final population pharmacokinetic model for cefaclor, relative standard error (RSE, %) values of all parameters estimated were 0.60–51.90%. Eta shrinkage (%) values of V/F, CL/F, and K_a_ were in the range of 0.069–0.384%, which were acceptable. IIV (%) values of V/F and CL/F were relatively low at 10.250 and 18.388%, respectively, lower than those of a previously established cefaclor base model (IIV values for V/F and CL/F in cefaclor’s base model were 13.772 and 22.238%, respectively). This indirectly implied that the final population pharmacokinetic model for cefaclor with CrCl and body weight as covariates for CL/F and V/F was appropriate. The correlation coefficient was 0.436 between CL/F and CrCl (dCL/FdCrCl). It was 0.581 between V/F and body weight (dV/FdWeight), showing positive relationships. These results were consistent with previous covariate search results (shown in Figure 2). In other words, the results of the covariate search that CL/F and V/F increased with the increasing CrCl and weight of individuals were well reflected in the final model of cefaclor’s population pharmacokinetics. The following results were predicted and suggested through the positive correlation between CL/F and CrCl in the population pharmacokinetic model of cefaclor identified in this study: As renal function progresses to mild, moderate, severe, and end-stage impairment, the values of creatinine clearance will decrease. As a result, as the CL/F of cefaclor gradually decreases, the amount of blood exposure will increase. However, the degree of exposure can be clearly confirmed only when cefaclor administration data are obtained for the patient group with renal function problems in the future. K_a_ related to oral absorption of cefaclor had a large ω^2^ estimate of 0.971 in the final model, with RSE of 30.55%. The IIV value was also high at 98.534. This suggests that oral absorption of cefaclor varies between individuals. Further studies with other potential covariates are needed to explain the variability of absorption of cefaclor. Based on results of this study, it is necessary to further study genetic factors related to oral absorption of cefaclor. As mentioned earlier, *PEPT1* could be a dominant covariate. The T_lag_ estimated by the model is 0.245 h, which is very close to the first sampling point in this study, 0.25 h. However, the oral absorption rate constant was as high as 5.203 h^−1^. In the future, more research is needed with more diverse populations and improved assays, but it may be thought that a certain delay time (although close to the time of the first sampling point) is required for the drug to be absorbed into the blood from the gastrointestinal tract and then quickly reach the blood. The reason for the delay in oral absorption of cefaclor may be related to dissolution of cefaclor from formulation, and transit through gastrointestinal tract. Of course, in order to clarify, it will be clearer than the present through the use of tighter sampling point settings (at absorption phase) and sensitive analysis method application.

The correlations between body weight and V/F (of cefaclor), CrCl and CL/F (of cefaclor) explored in this study were results that were not clearly confirmed in previous studies (referred to Tables S1 and S2). Although this population pharmacokinetic model study was conducted only on healthy adults, it was nevertheless very important and interesting to find significant correlations between body weight and V/F, creatinine clearance and CL/F. This is because, with respect to the covariates explored in this study, it implies the possibility that the pharmacokinetic correlation will be more significantly confirmed (than this study) in the future in patients with kidney disease or in the obese group. Therefore, if pharmacokinetic modeling is additionally performed for more diverse groups based on this study, the correlation with the derived covariates could be a strong basis for suggesting that it needs to be reflected in the actual clinical application of cefaclor.

There are continuous interest and attempts of formulation research for cefaclor. The current cefaclor capsule has a very short T_1/2_ in the blood (within 1 h) while the CL/F is very large. To improve the clinical effect and improve these limitations, there are attempts to make cefaclor nanoparticles or sustained release formulations using polymers. We believe that this study will be a useful reference for the development and clinical application of new formulations of cefaclor in the future because cefaclor’s pharmacokinetics can be of great help in predicting and interpreting its clinical results by providing information on the degree and relevance of renal function and weight. Results of this study can also be used as evidence for initial clinical dose in an administration setting.

The limitation of this study is that a population pharmacokinetic modeling analysis was performed with pharmacokinetic results on healthy male Koreans (as a limited group). That is, cefaclor pharmacokinetic studies were conducted on young men (average 23 years old). In the future, pharmacokinetic studies will need to be carried out in more diverse groups, namely women, elderly and obese groups.

### 3.5. Population Pharmacokinetic Model Evaluation

**Figure 4:**
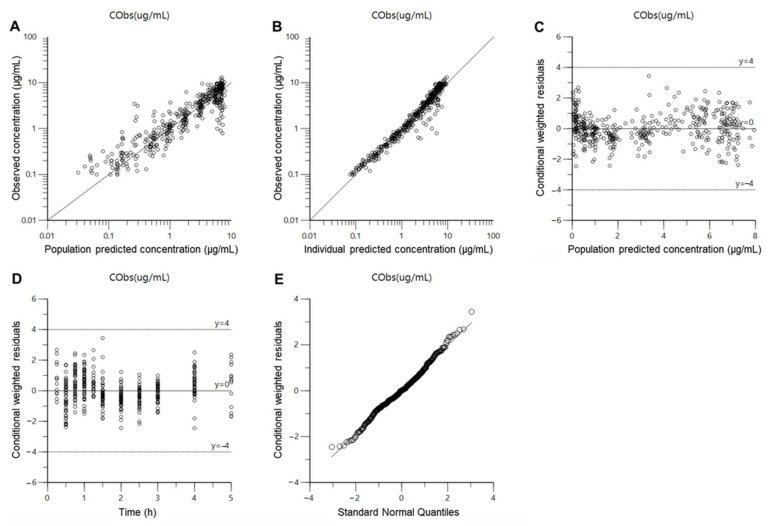
Goodness-of-fit plots of the final population pharmacokinetic model for cefaclor. (**A**) Population-predicted concentrations (PRED) against observed plasma concentration (DV); (**B**) individual-predicted concentrations (IPRED) against DV; (**C**) PRED against conditional weighted residuals; (**D**) time (IVAR) against conditional weighted residuals; (**E**) quantile–quantile plot of components of conditional weighted residuals.

The established cefaclor population pharmacokinetic model was comprehensively evaluated for goodness-of-fit plots, visual predictive check, bootstrapping, and normalized prediction distribution error. Figure 4 shows results of a goodness-of-fit plot for the final population pharmacokinetic model of cefaclor. Cefaclor concentrations predicted with the population pharmacokinetic model for the population and individuals showed relatively good agreement with experimentally obtained observations. Conditional weighted residuals were well distributed symmetrically with respect to zero. That is, conditional weighted residuals were well distributed at random without any specific bias. Conditional weighted residual values at all points of predicted concentration or time in the population did not deviate from ±4. The quantile–quantile plot of components of conditional weighted residuals was close to a straight line where *x*- and *y*-axes were symmetrical. Goodness of fit plot results presented in Figure 4 suggested that the final established population pharmacokinetic model of cefaclor had no graphically significant problems.

Table 6 shows bootstrapping results for the final population pharmacokinetic model established for cefaclor. All the parameter values estimated with this final model were within 95% confidence intervals of bootstrap analysis results (number of replication: 1000). Estimated values of model parameters were almost similar to the median estimated by bootstrap analysis. Results of bootstrapping analysis confirmed the robustness and reproducibility of the final population pharmacokinetic model established for cefaclor. 

**Figure 5:**
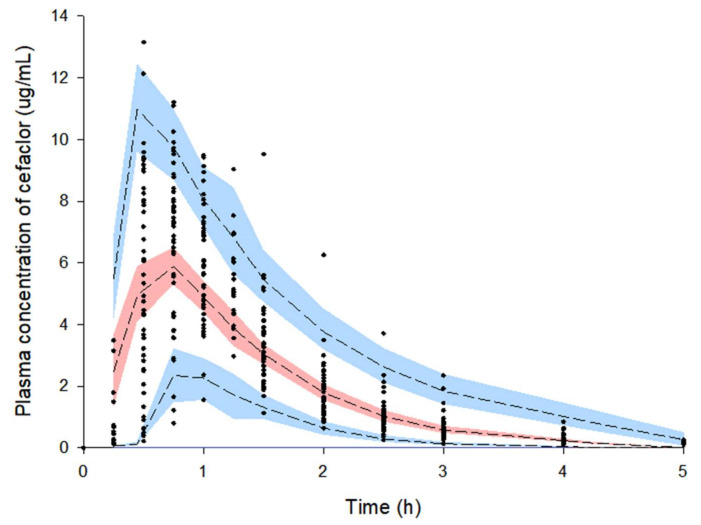
Visual predictive check of the final model for cefaclor. Observed concentrations were depicted by dots. Black dashed lines indicate 95th, 50th, and 5th percentiles of predicted concentrations. Blue shaded regions indicate 95% confidence intervals for predicted 5th and 95th percentiles. Red shaded regions indicate 95% confidence intervals for the predicted 50th percentiles.

Figure 5 shows results of a visual predictive check for the final population pharmacokinetic model established for cefaclor. Most observation values of cefaclor were well distributed within 90% prediction intervals (of 5–95%) of prediction values. Visual predictive check results suggested that the population pharmacokinetic model of cefaclor described overall experimental data relatively well.

**Figure 6:**
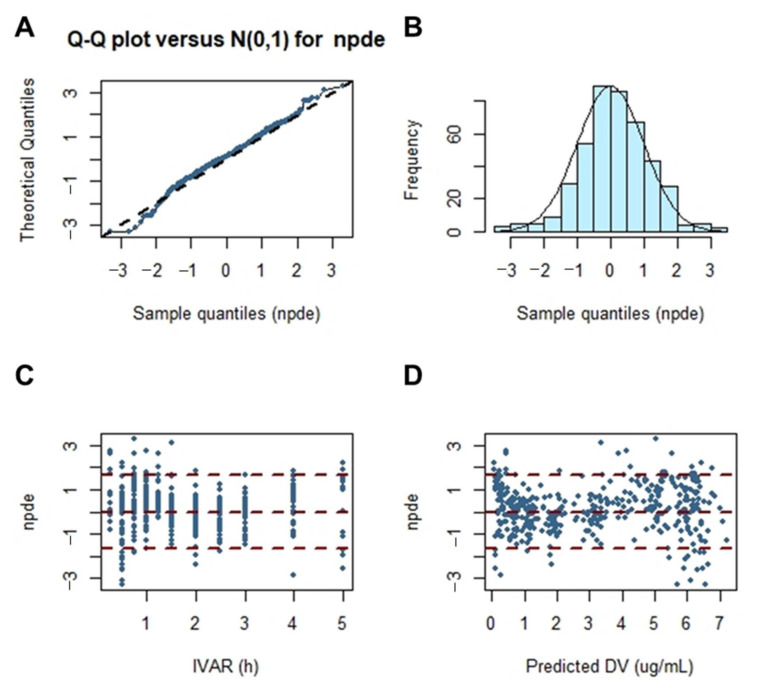
Normalized prediction distribution error for the final model. Quantile–quantile plots of normalized prediction distribution error versus theoretical *n* (0, 1) distribution (**A**). Histogram showing the distribution of normalized prediction distribution error overlaid with density of the standard Gaussian distribution (**B**). Scatter plot of time versus normalized prediction distribution error (**C**). Scatterplot of predictions versus normalized prediction distribution error (**D**).

Figure 6 shows the results of the normalized prediction distribution error analysis for the final population pharmacokinetic model established for cefaclor. Normalized prediction distribution error is an analysis that considers the overall predicted distribution of each individual observation and processes multiple observations within a subject. Thus, it was necessary to verify the normality through normalized prediction distribution error analysis. The assumption of a normal distribution for differences between predictions and observations was acceptable. The quantile–quantile plots and histograms also confirmed the normality of the normalized prediction distribution error. Results of a comprehensive evaluation of the final established population pharmacokinetic model of cefaclor were all acceptable without major problems.

## 4. Conclusions

In this study, a human population pharmacokinetic model for cefaclor was developed. To the best of our knowledge, this is the first report on cefaclor population pharmacokinetics in Koreans using pharmacokinetic data of healthy subjects. Plasma concentration–time profiles for cefaclor were well explained by a one-compartment model of first-order absorption and elimination with absorption lag-time. CrCl and body weight were identified as effective covariates of CL/F and V/F of cefaclor, respectively. Their correlations were reflected in the final population pharmacokinetic model of cefaclor. The results of this study are expected to serve as an important reference for model improvement along with the search for other valid covariates of cefaclor in the future. In addition, it is expected that treatment effect can be improved while reducing side effects by adjusting the dose and dosage of cefaclor that reflect CrCl, an index of renal function. Although it is known that cefaclor is not an essential drug for therapeutic drug monitoring, studies on dose and usage for effective treatment in a variety of groups than before through population pharmacokinetic modeling studies of cefaclor are required. This is because, so far, no clear exploratory studies of effective covariates related to the intra-population pharmacokinetic diversity of cefaclor have been performed and reported. Furthermore, despite the long clinical use of cefaclor, it is being applied to various groups (collectively) as an empirical therapy without a clear scientific basis. Through the population pharmacokinetic modeling study of cefaclor, the search for effective covariates related to the pharmacokinetic diversity of cefaclor can be made, and the optimal dose and usage studies taking this into account (based on this study) will be an opportunity to continue in the future.

## Figures and Tables

**Table 1:** Demographic information of study subjects (*n* = 48).

Physicochemical Parameters	Units	Median (Range)	Mean ± SD
Age	Year	23 (19–26)	23.00 ± 1.44
Weight	kg	66.05 (50.00–88.70)	67.31 ± 8.46
Height	cm	172.0 (164.00–187.30)	173.31 ± 6.22
BSA *	m^2^	1.78 (1.51–2.10)	1.80 ± 0.13
BMI **	kg/m^2^	21.78 (17.70–28.44)	22.40 ± 2.44
Albumin	g/dL	4.90 (4.40–5.40)	4.85 ± 0.23
Total proteins	g/dL	7.40 (6.70–8.20)	7.38 ± 0.34
BUN	mg/dL	12.00 (7.70–21.80)	12.54 ± 3.39
Total bilirubin	mg/dL	1.00 (0.51–2.11)	1.04 ± 0.34
Cholesterol	mg/dL	158 (118–231)	163.52 ± 29.70
ALT	U/L	15.00 (7.00–41.00)	16.42 ± 6.70
AST	U/L	17.00 (6.00–28.00)	17.29 ± 3.96
ALP	U/L	74.00 (44.00–105.00)	73.92 ± 16.77
Creatinine	mg/dL	1.00 (0.70–1.30)	0.97 ± 0.12
CrCl ***	mL/min	110.92 (67.50–170.42)	114.97 ± 20.86

* BSA was calculated using the Mosteller equation as follows: $\sqrt{\left(\mathrm{height}\;\left(\mathrm{cm}\right)\times \;\mathrm{weight}\;\left(\mathrm{kg}\right)/3600\right)}$ [9]. ** BMI was calculated as the Kaup index as follows: body weight (kg)/height^2^ (m^2^) [10]. *** CrCl was calculated with the Cockcroft–Gault equation as follows: [(140 − age) × body weight (kg)]/[serum creatinine (mg/dL) × 72] [11].

**Table 2:** Estimated pharmacokinetic parameters of cefaclor by non-compartmental analysis after a single oral administration of a 250 mg cefaclor capsule (mean ± SD, *n* = 48).

Parameters	Units	Estimates
AUC_0–t_	h·µg/mL	9.64 ± 2.40
AUC_0–∞_	h·µg/mL	9.83 ± 2.41
CL/F	mL/h	27,188.46 ± 7771.88
C_max_	μg/mL	7.87 ± 2.20
T_1/2_	h	0.68 ± 0.15
T_max_	h	0.80 ± 0.28
V/F	mL	26,116.39 ± 7820.88

**Table 3:** Base structural model building steps.

Model	Description	*n*–Parameter	−2LL	AIC	Δ−2LL	ΔAIC	Compared with
**Absorption model**							
01	1–compartment with first order (without T_lag_)	7	1626.01	1640.01	−	−	−
02 *	1–compartment with first order (with T_lag_)	9	1019.51	1037.51	−606.50	−602.50	01
**Residual error model**							
02	Additive	9	1019.51	1037.51	0	0	02
02–01 *	Proportional	9	882.99	900.99	−136.52	−136.52	02
02–02	Power	9	1109.68	1127.68	90.17	90.17	02
02–03	Mixed	10	1493.82	1511.82	474.31	474.31	02
02–04	Log additive	9	1493.82	1511.82	474.31	474.31	02
**IIV model**							
02–01–01	Remove IIV V/F	8	1772.84	1788.84	889.85	887.85	02–01
02–01–02	Remove IIV CL/F	8	1092.38	1108.38	209.39	207.39	02–01
02–01–03	Remove IIV Ka	8	1570.07	1586.07	687.08	685.08	02–01
02–01–04 *	Remove IIV T_lag_	8	859.92	875.92	−23.07	−25.07	02–01
02–01–05	Remove IIV T_lag_ and CL/F	7	1076.22	1090.22	193.23	189.23	02–01

* selected models at each step.

**Table 4:** Stepwise search for covariates.

Model	Description	OFV	ΔOFV	*n*–Parameter	Compared with
01	Base model	859.918	−	8	−
02	Weight on CL/F	857.838	−2.080	9	01
03	BSA on CL/F	857.163	−2.755	9	01
04	BMI on V/F	857.087	−2.831	9	01
05	Weight on V/F	855.211	−4.707	9	01
06	CrCl on CL/F	855.157	−4.761	9	01
07	CrCl and BSA on CL/F	855.276	0.119	10	06
08 *	CrCl on CL/F and weight on V/F	848.495	−6.662	10	06

* selected final model.

**Table 5:** Estimated population pharmacokinetic parameters for cefaclor with the final model.

Parameters	Units	Estimate	SE	RSE (%)	Shrinkage (%)	IIV (%)
tvV/F	mL	22,593.260	790.037	3.50	−	−
tvCL/F	mL/h	27,166.883	981.584	3.61	−	−
tvT_lag_	h	0.245	0.001	0.60	−	−
tvK_a_	1/h	5.203	0.938	18.02	−	−
dCL/FdCrCl	−	0.436	0.184	42.21	−	−
dV/FdWeight	−	0.581	0.183	31.54	−	−
ω^2^_V/F_	−	0.011	0.005	51.90	0.384	10.250
ω^2^_CL/F_	−	0.034	0.010	30.22	0.069	18.388
ω^2^_Ka_	−	0.971	0.297	30.55	0.079	98.534
σ	−	0.270	0.023	8.56	−	−

SE, standard error; RSE, relative standard error.

**Table 6:** Estimated population pharmacokinetic parameter values of cefaclor and bootstrap validation (*n* = 1000).

Parameters	Units	Final Model		Bootstrapping	
		Estimate	95% Confidence Interval	Median	95% Confidence Interval
tvV/F	mL	22,593.260	21,040.330–24,146.190	22,571.198	20,976.447–24,091.153
tvCL/F	mL/h	27,166.883	25,237.440–29,096.327	26,952.524	25,315.334–29,579.444
tvT_lag_	h	0.245	0.242–0.248	0.245	0.240–0.247
tvK_a_	1/h	5.203	3.360–7.046	5.198	3.806–10.828
dCL/FdCrCl	−	0.436	0.074–0.798	0.436	0.125–0.870
dV/FdWeight	−	0.581	0.221–0.941	0.581	0.248–0.920
ω^2^_V/F_	−	0.011	0.000–0.021	0.009	0.000–0.023
ω^2^_CL/F_	−	0.034	0.014–0.054	0.031	0.010–0.052
ω^2^_Ka_	−	0.971	0.390–1.552	0.962	0.369–1.555
σ	−	0.270	0.225–0.316	0.268	0.215–0.311

## Data Availability

The data presented in this study are available in the article and Supplementary Materials.

## Supplementary Materials

The following are available online at https://www.mdpi.com/article/10.3390/pharmaceutics13050754/s1, Figure S1. Relationship between subjects’ characteristics and individual predicted pharmacokinetic parameters. V/F of cefaclor according to BMI (A), V/F of cefaclor according to BSA (B), V/F of cefaclor according to total protein (C), CL/F of cefaclor according to BUN (D), CL/F of cefaclor according to ALT (E), and CL/F of cefaclor according to BMI (F); Table S1. Previously reported pharmacokinetic parameter values of cefaclor in Koreans obtained by non-compartmental analysis; Table S2. Previously reported pharmacokinetic parameter values of cefaclor in non-Koreans.

## Abbreviations

CrCl	Creatinine clearance
BSA	Body surface area
BMI	Body mass index
AST	Aspartate transaminase
ALP	Alkaline phosphatase
ALT	Alanine transaminase
HPLC	High–performance liquid chromatography
UV	Ultraviolet
SD	Standard deviation
NLME	Nonlinear mixed effects
AIC	Akaike’s information criterion
IIV	Inter–individual variability
OFV	Objective function value
SE	Standard error
RSE	Relative standard error
