# Supplementary material for: Population Pharmacokinetic Analysis of Cefaclor in Healthy Korean Subjects
Source: Pharmaceutics. 2021 May 19;13(5):754. doi: 10.3390/pharmaceutics13050754 (PMC8160640; doi:10.3390/pharmaceutics13050754)
Supplement: Supplementary file 1 [file pharmaceutics-13-00754-s001.zip › pharmaceutics-1206518 final supplementary.pdf]

pharmaceutics

Supplementary Materials: Population Pharmacokinetic Analysis of Cefaclor in Healthy Korean Subjects

Seung–Hyun Jeong, Ji–Hun Jang, Hea–Young Cho and Yong–Bok Lee

Supplementary tables

Table S1. Previously reported pharmacokinetic parameter values of cefaclor in Koreans obtained by non-compartmental analysis.

References	Subjects	Pharmacokinetic parameters				
		$T_{1/2}$ (h)	T_{max} (h)	C_{max} (μg/mL)	$AUC_{0-\infty}$ (h·μg/mL)	CL/F (L/h)
Yun et al. (2002) [15]	Human (*n* = 20, 250 mg dose, male)	1.29–1.32	0.83–0.90	5.00–5.11	6.88–7.14	35.31–36.00
Chun et al. (2002) [16]	Human (*n* = 16, 500 mg dose, male)	-[a]	0.83–0.89	12.17–13.03	15.76–17.65[b]	-[a]
Cho et al. (2005) [12]	Human (*n* = 24, 250 mg dose, male)	-[a]	0.76–0.82	6.72–6.90	7.73–7.88[b]	-[a]
Kim et al. (2005) [17]	Human (*n* = 24, 250 mg dose, male)	-[a]	0.72–0.79	5.36–5.43	6.60–6.67[b]	-[a]
In this study	Human (*n* = 48, 250 mg dose, male)	0.68 ± 0.15	0.80 ± 0.28	7.87 ± 2.20	9.83 ± 2.41	27.19 ± 7.77

[a], information not provided in those reports. [b], AUC_{0-t} provided in those reports.

Table S2. Previously reported pharmacokinetic parameter values of cefaclor in non-Koreans.

References	Subjects (healthy humans)	Dose	Pharmacokinetic parameters				
			$T_{1/2}$ (h)	T_{max} (h)	C_{max} (μg/mL)	$AUC_{0-\infty}$ (h·μg/mL)	CL/F (L/h)
Bloch et al. (1977) [5]	*n* = 25, Male in USA	250 mg	0.7–1	-[a]	-[a]	-[a]	-[a]
Spyker et al. (1978) [4]	*n* = 24, Male in USA	500 mg	-[a]	0.88 ± 0.33	23.1 ± 7.7	-[a]	-[a]
Lode et al. (1979) [18]	*n* = 12, Male = 6, Female = 6 in Germany	1,000 mg	-[a]	-[a]	34.6 ± 7.8	74.5 ± 9.9	-[a]
Welling et al. (1979) [19]	-[a]	500 mg	-[a]	1–1.5	-[a]	-[a]	-[a]
Barbhaiya et al. (1990) [20]	*n* = 12, Male in USA	250 mg	0.5 ± 0.2	0.5 ± 0.0	10.6 ± 2.4	8.7 ± 1.4	10.92 ± 6.54[b]
Barbhaiya et al. (1990) [21]	*n* = 12, Male in USA	250 mg	0.83 ± 0.21	0.4–1.0	8.70 ± 2.72	8.60 ± 1.43	-[a]
Barbhaiya et al. (1990) [22]	*n* = 12, Male in USA	500 mg	0.54 ± 0.10	-[a]	16.7 ± 3.67	16.9 ± 2.5	22.62 ± 3.68[b]

Oguma et al. (1991) [26]	*n* = 8, Male in Japan	500 mg	0.59	-[a]	-[a]	18.6[c]	-[a]
Nix et al. (1997) [23]	*n* = 12, Male = 7, Female = 5 in USA	500 mg	0.69 ± 0.17	0.92 ± 0.43	15.9 ± 5.72	20.6 ± 3.38	24.9 ± 4.38
Sourgens et al. (1997) [25]	-[a]	-[a]	0.5–0.7	0.5–1	-[a]	-[a]	-[a]
Granados-Soto et al. (2003) [24]	*n* = 6, Male in Mexico	500 mg	-[a]	0.87 ± 0.06	11.45 ± 1.6	14.93 ± 1.64	-[a]
Li et al. (2009) [7]	*n* = 18, Female in USA	500 mg	0.59 ± 0.09	0.5–2	13.4 ± 3.5	21.2 ± 4.2	23.82 ± 4.96

[a], information not provided in those reports. [b], information provided as renal clearance (CL_R). [c], AUC_{0-t} provided in those reports.

Supplementary figure

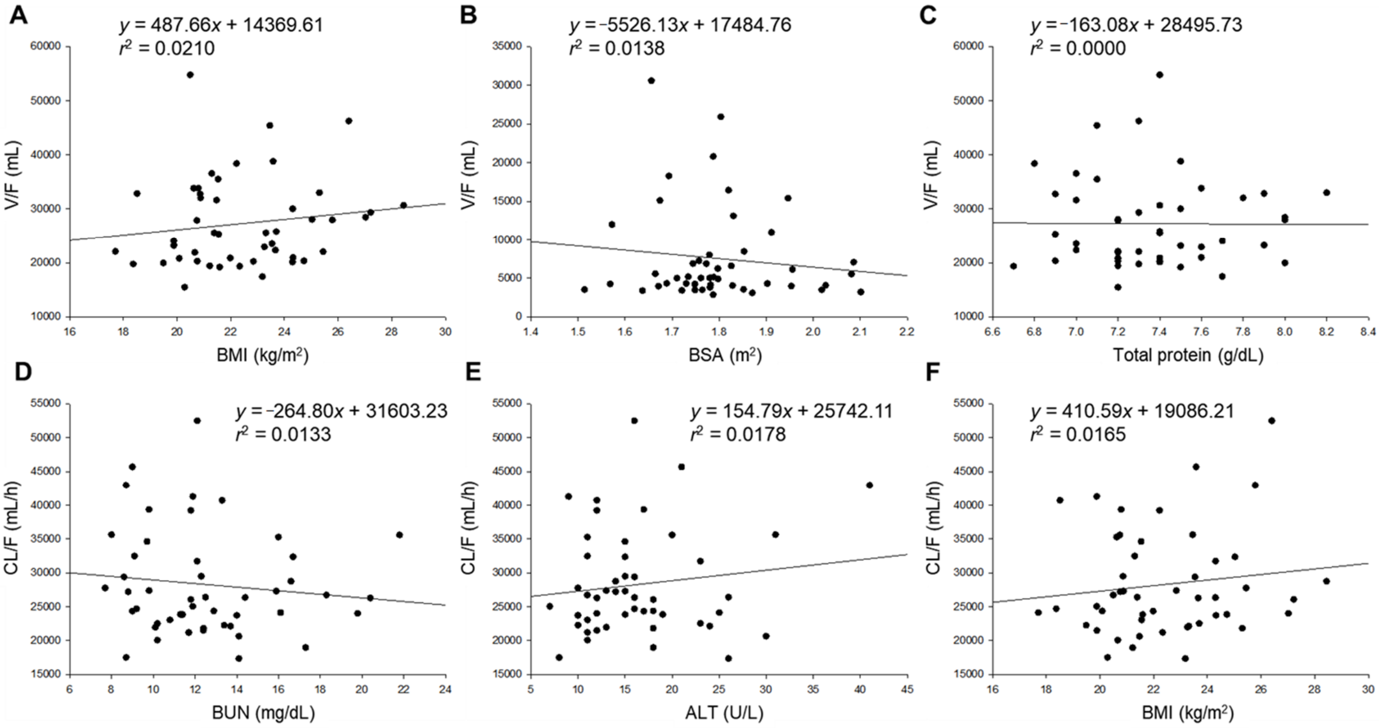

Figure S1. Relationship between subjects' characteristics and individual predicted pharmacokinetic parameters. V/F of cefaclor according to BMI (A), V/F of cefaclor according to BSA (B), V/F of cefaclor according to total protein (C), CL/F of cefaclor according to BUN (D), CL/F of cefaclor according to ALT (E), and CL/F of cefaclor according to BMI (F).
